# Microalgal cultivation characteristics using commercially available air-cushion packaging material as a photobioreactor

**DOI:** 10.1038/s41598-023-30080-6

**Published:** 2023-03-07

**Authors:** Clifford R. Merz, Neha Arora, Michael Welch, Enlin Lo, George P. Philippidis

**Affiliations:** 1 College of Marine Science, University of South Florida, St. Petersburg, FL USA; 2 Department of Cell, Microbiology and Molecular Biology, University of South Florida, Tampa, FL USA; 3 Patel College of Global Sustainability, University of South Florida, Tampa, FL USA

**Keywords:** Biofuels, Plant biotechnology, Biochemistry, Biotechnology, Biogeochemistry, Engineering

## Abstract

Air-cushion (AC) packaging has become widely used worldwide. ACs are air-filled, dual plastic packaging solutions commonly found surrounding and protecting items of value within shipping enclosures during transit. Herein, we report on a laboratory assessment employing ACs as a microalgal photobioreactor (PBR). Such a PBR inherently addresses many of the operational issues typically encountered with open raceway ponds and closed photobioreactors, such as evaporative water loss, external contamination, and predation. Using half-filled ACs, the performance of microalgal species *Chlorella vulgaris, Nannochloropsis oculata*, and *Cyclotella cryptica* (diatom) was examined and the ash-free dry cell weight and overall biomass productivity determined to be 2.39 g/L and 298.55 mg/L/day for *N. oculata*, 0.85 g/L and 141.36 mg/L/day for *C. vulgaris,* and 0.67 g/L and 96.08 mg/L/day for *C. cryptica.* Furthermore, maximum lipid productivity of 25.54 mg/L/day AFDCW and carbohydrate productivity of 53.69 mg/L/day AFDCW were achieved by *C. cryptica*, while maximum protein productivity of 247.42 mg/L/day AFDCW was attained by *N. oculata*. Data from this work will be useful in determining the applicability and life-cycle profile of repurposed and reused ACs as potential microalgal photobioreactors depending upon the end product of interest, scale utilized, and production costs.

## Introduction

Human activities, such as overconsumption and overpopulation, have contributed to environmental deterioration of the biophysical environment through resource depletion, ecosystem and habitat destruction, and pollution. This includes, but is not limited to, wild-capture fish stock depletion and increasing levels of ocean plastic pollution. Mitigation of these deleterious effects will require a sustainable development based on a water-food-energy nexus methodology that recognizes the interconnectivity of environmental resources, capacity, and waste. Pursuant to this, the 4R sustainability concepts of Reduce, Reuse, Recycle, and Repurpose (rethink) are being incorporated more frequently into environmental life cycle assessment (LCA) approaches, especially those that formulate plastics waste management strategies and public policy^1^. Although these 4Rs of sustainability are not new concepts, never have they been more important than today in a world whose estimated 2050 population of 9.8 billion reflects an increase of nearly 2 billion people over the most recent 30-year period. Presuming the population trends play out, the production of microalgae will be increasingly needed to ensure a sufficient supply of nutritious protein and other bioproducts to the world’s population^2,3^.

Over the years, many microalgae technoeconomic analyses have been published focusing on greenhouse gas (GHG) balances^4^ and water use balances^5,6^. Although significant interest and effort in large-scale cultivation of algae has been explored for decades, the industry is still plagued by high production costs necessitating the development of innovative ideas in cultivation and harvesting methodologies^7^. Microalgae are unicellular organisms commonly found in nature prospering under many different environmental conditions. Some of these environments can be quite hostile, resulting in the internal development of survival mechanisms to counteract environmental stressors. Many of these cellular stress responses result in the biosynthesis of products of commercial interest, such as antioxidants and carotenoids under intense ultraviolet light exposure, along with general metabolites and other functional nutrients^8^. Commercial production of microalgae is currently accomplished using a variety of cultivation designs, including raceway ponds (RWP), tubes, polyethylene plastic bags, indoor and outdoor photobioreactors (PBRs), and fermenters^9–12^. Often, outdoor axenic microalgae cultures begin with inoculum grown indoors, where predators are excluded and nutrients and environmental conditions are controlled, and is then moved to outdoor tanks or raceways for mass production^3^.

Results from detailed “cradle to grave” assessments will reveal appropriate R&D development and project improvement strategies for innovative alternatives that arise. One such microalgal biorefinery alternative is Bubble Farming (BF), where sheets of plastic bubble wrap are envisioned to be manufactured on-location using modified farm equipment and then placed onto the land surface with each bubble filled with a specific aqueous algal mixture for later harvest^13^. Sheet and individual bubble diameters are sized to fit a specific region’s growing season and terrain, anticipated application, and desired end use, such as Diafuel (biofuel from diatoms)^14^. BF inherently addresses many of the operational issues typically encountered with open RWP and closed PBR systems, such as evaporative water loss, external contamination, and predation. Another microalgal biorefinery alternative is the plastic OMEGA bioreactor developed by NASA^15^ for microalgal wastewater remediation deployed close to offshore aquaculture cages, nearshore to coastal communities in close proximity to on-land family/agribusiness sized aquaculture farms (enabling use of non-arable or fallowed land and BF/crop rotation) or offshore outfall wastewater disposal. OMEGA is a system for cultivating microalgae using wastewater contained in large, floating, linear low-density polyethylene PBRs deployed in marine environments, which employs forward osmosis across tubular membrane walls to passively concentrate nutrients and dewater the microalgal biomass for later conversion to biofuels or fertilizers^16^.

In the present study, microalgal cultivation characteristics were assessed using a reused and repurposed plastic air-cushion (AC) packing material as photobioreactor. ACs are dual-material custom packaging solutions usually fabricated from polyethylene and shipped filled with air. ACs are commonly found surrounding and protecting items of value within many shipping enclosures during transit. The typical AC consists of a thin plastic film that is recyclable, but not necessarily biodegradable. Consumer shift from using bubble wraps to air cushions for packing has been observed in recent years mainly due to their perceived sustainability and ease of use. ACs also take up less space in warehouses, since they are typically filled with air upon usage, thus reducing storage cost, improving use of space, and allowing reuse in subsequent shipments. In fact, the global AC packaging market size is projected to reach $4.4 billion by 2025 registering a compound annual growth rate of 7.0%^17^. This global growth in demand is attributed to a rising need for protection of goods from harsh transit conditions and surfaces.

Given the expansion of AC production, it is important to recycle or repurpose the used or excess material in order to reduce and mitigate resulting plastic waste. One possible repurposed use for ACs is microalgae cultivation. Repurposed and reused ACs can help reduce capital investment in the microalgal biorefinery enterprise, while providing an isolated, contaminant-free environment during cultivation. Moreover, sealed ACs inherently address many of the operational issues typically encountered with traditional open RWP and closed PBR microalgal bioproduction, such as evaporative water loss, external contamination, and predation. Pursuant to this, to the best of our knowledge, a first laboratory assessment of microalgal cultivation and biomass and lipid production by three microalgal species (freshwater and marine), namely *Chlorella vulgaris* UTEX 395*, Nannochloropsis oculata* CCMP 525, and diatom *Cyclotella cryptica* CCMP 332^18^, was conducted to determine whether commercially available and frequently discarded ACs can serve as PBRs without modification and at what level of productivity.

The methodology used in this study focused initially on a qualitative approach to determine if positive CO_2_ and O_2_ gas exchange occurs across the AC’s non-specially formulated plastic material. Followed by a quantitative approach wherein physiological changes in the algae cultivated in AC-PBRs under the same abiotic conditions [light, CO_2_ (aeration/gas exchange), pH, temperature, volume] were determined by analyzing growth, biochemical composition, and fatty acid profiles. Additionally, *C. cryptica* testing was performed to elucidate the effects of the biogenic silica, which included Ash-Free Dry Weight and nitrogen elemental analysis. Finally, a LC–MS (liquid chromatography-mass spectroscopy) analysis was performed to determine the synthesis of the high-value carotenoid fucoxanthin by the diatom *C. cryptica*. Data from this assessment will be useful in the determination of the conceptual applicability and utility of ACs as a potential stand-alone microalgal PBR, as well as in LCA techno-economic analyses, with the ultimate economic and feasibility decisions depending upon the value of the target end product (biomass, lipid, protein or carbohydrate) developed and the scale utilized.

## Materials and methods

### Microalgae cultivation

*Nannochloropsis oculata* CCMP 525 (henceforth *N. oculata*) and *Cyclotella cryptica* CCMP 332 (henceforth *C. cryptica*) were procured from the National Center for Marine Algae and Microbiota (NCMA) (Bigelow laboratory for Ocean Sciences, Maine, USA). *N. oculata* was maintained in macronutrient modified F/2 (Phyto Technology Laboratories, USA) marine medium. *C. cryptica* was maintained in macronutrient modified L1 (NCMA) marine medium. *Chlorella vulgaris* UTEX 395 (henceforth *C. vulgaris*) was purchased from the culture collection of algae at the University of Texas (Austin, USA) and maintained in Bold’s Basal Medium (BBM, Phyto Technology Laboratories, USA). The pH of all the media was set at 7.5 using a pH meter (Orion 3 Star, Thermo Fisher, USA). For inoculum preparation, the algal strains were cultivated in the respective media using 125-mL Erlenmeyer flasks with a working volume of 50 mL under continuous white light with an intensity of 100 µmol/m^2^s in an incubator shaker (Excella E24, New Brunswick Scientific, Eppendorf, Germany) at 150 rpm at 23 °C for 3–4 days (log phase).

### Repurposed and reused AC microalgal photobioreactor: preliminary experiments

The AC-PBR selected for all three microalgal species examined herein was a commercially available AC packaging material typically found in shipping boxes. To ensure consistency during the study, a sufficient number of air-filled AC units was acquired (AIRplus by STOROpack), each measuring 90 mm × 180 mm × 0.0254 mm (3.543 × 7.087 × 0.001 inch) and made of clear low-density polyethylene (LDPE). Some of these ACs were kept on-site for research discussed herein and some were provided to colleagues at Dr. Hari Singh Gour University in India for their research investigations^19^. Each sourced AC-PBR can theoretically hold ~ 380 mL of liquid. Although preliminary experiments run at 1/4, 1/2, and 3/4 capacity revealed approximately equivalent optical density (O.D._680 nm_) results across the algal species tested, the most consistent results were obtained at half capacity (Supplementary data). Therefore, the 1/2 filled (~ 190 mL) AC-PBR configuration was selected in this study.

#### *CO*_*2*_* and O*_*2*_* gas exchange across the AC-PBR surface*

The first step in determining if growth will in fact occur and at what level of productivity was to ascertain whether CO_2_ and O_2_ gas exchange would occur across the AC-PBR surface. Qualitative findings of the CO_2_ and O_2_ Gas Exchange across the AC-PBR surface are reported in “Determining gas exchange in AC-PBR” section.

CO_**2**_: Two AC-PBRs were filled with compressed pure CO_2_ gas supplied by Airgas (USA), heat sealed, and submerged underwater to confirm no leakage. One AC-PBR (initially filled to 250 mL) was placed on the lab tabletop at 23 °C to observe if CO_2_ diffused across the LDPE plastic into the atmosphere overnight under a positive gradient. The second AC-PBR (initially filled to 300 mL with CO_2_) was submerged in a covered container of water at an initial pH of 7.1. The total volume within the container was 5.5 L with no bubbles observed escaping from the AC-PBR nor attached to the surface.

O_2_: Two AC PBRs were filled with compressed pure O_2_ gas supplied by Bernzomatic (USA), heat sealed, and submerged underwater to confirm no leakage. One AC-PBR was placed on the lab tabletop to visually observe if O_2_ diffused across the LDPE plastic into the atmosphere under a positive gradient overnight. The second AC-PBR was submerged in a covered water container with a total volume of 3.0 L with no bubbles observed to be escaping from the AC-PBR nor attached to the surface.

#### *CO*_*2*_* addition*

Most microalgal investigations describe PBRs with some form of active and continuous delivery of filtered compressed CO_2_ gas as a carbon source. Although the AC CO_2_ gas exchange results were promising, a concern remained as to whether enough CO_2_ would be present throughout the cultivation period simply by gas exchange across the AC-PBR surface. A preliminary test of *C. vulgaris* revealed that while the microalgae remained visually green, growth as measured by O.D._680 nm_ only increased by 10.8% over the 10-day test period without additional CO_2_ supplementation. To enhance the growth of the algal strains, we added sodium bicarbonate (NaHCO_3_ = 48 g/L) to both *C. vulgaris* and *N. oculata* AC-PBRs. This procedure was further modified by initially bubbling in filtered compressed CO_2_ gas until solution saturation was reached (i.e., dropping pH stabilized) followed by the titration of the media with saturated sodium bicarbonate solution to a pH of 7.

#### Light intensity

Proper light intensity is an important parameter to ensure that the energy source required for photosynthesis passes not only through the AC-PBR plastic surface, but also penetrates far enough inside to reduce any cell-to-cell shading effects^20^. A portable light meter (Model CA813, AEMC Instruments) was used to measure the light intensity in lumens per meter (lux) at the surface of the AC PBR. Test runs were conducted on *C. vulgaris and N. oculata* under three conditions: (1) One light (Airand CT-X01A600-18 Natural White LED lights) at 77 µmol/m^2^s, no shaking with 16:8 h (light:dark) photoperiod; (2) One light on a shaker table at 75 rpm and 65 µmol/m^2^s with 24-h (continuous) illumination; and (3) Two lights at a total 180 µmol/m^2^s with a 16:8-h (light:dark) photoperiod. Initial results using non-CO_2_ enhanced medium revealed no significant O.D. differences between shaker table and non-shaker one-light configuration, but two-light non-shaker results were slightly better than the one-light configuration (data not shown). Therefore, two rows of LED lights were selected as the indoor baseline lighting test configuration.

### Experimental setup

In support of the goal of determining whether cell growth will in fact occur within the AC- PBR and at what level of productivity, two runs with each of the three selected microalgal species were conducted in the respective media. An initial inoculum (O.D._680 nm_ = 0.6–0.8) of 10% (v/v) was used for all the species examined. Once suitable cellular O.D. was achieved in the shaker culture flasks, each algal suspension was harvested and added to freshly prepared medium, as described in “Microalgae cultivation” section. Prior to addition of the algae, the medium was enhanced by bubbling in filtered compressed CO_2_ gas until solution saturation was reached (i.e., dropping pH stabilized) followed by addition of saturated sodium bicarbonate (NaHCO_3_ = 48 g/L) until neutral pH was achieved. The final volume was increased stoichiometrically with macronutrient solution based on the amount of added NaHCO_3_.

Before AC-PBR test commencement, each air-filled AC-PBR was placed under a UV-C germicidal lamp for 15 min to sterilize the interior volume and prevent contamination. Through a small opening in the AC, approximately 190 mL (working volume) of algal cell suspension were pipetted inside with any extra air removed, and the AC-PBR (total volume 380 mL) was resealed using a VacMaster Pro110 external vacuum sealer machine. The system was checked for leaks.

The AC-PBRs for each algal strain was kept under two rows of LED with light intensity of 180 µmol/m^2^s with a 16:8-h (light:dark) photoperiod, no shaking at 25 °C. The cultivation was carried till each alga reached early stationary phase (*C. vulgaris*- 6 days, *N. oculata*- 8 days and *C. cryptica*-7 days). Photos of the AC-PBR microalgal cultivation test set-up for each strain can be found in Supplementary Data.

### Determination of algal growth, biochemical composition, fatty acid profile, and fucoxanthin

#### Determination of algal growth and nutrient consumption

Algal growth in terms of optical density at 680 nm (O.D._680 nm_) using a Spectrophotometer (DU 730, Beckman Coulter, Brea, CA, USA) and dry cell weight (DCW, g/L) was evaluated in duplicates for each algal species^21,22^. Every 24 h the O.D. was measured for each of the algae to derive a growth curve. Nitrate (NO_3_^−^), nitrite (NO_2_^−^), phosphate (PO_4_^−^), and pH were measured every 24 h using test strips (Hach Company, Loveland, Colorado, USA, and Micro Essential Laboratory (HYDRION), Brooklyn, New York, USA). At the end of the cultivation, the algal cells were harvested by centrifuging at 5000 g for 10 min at 25 °C and washed twice with distilled water (*C. vulgaris*) or 0.9% NaCl solution (*N. oculata* and *C. cryptica*) to remove media components. The resultant biomass was then placed in a 50 °C-oven to dry overnight. The DCW was gravimetrically determined using a benchtop digital scale (Mettler Toledo, USA) in accordance with the following equation:

$${\text{DCW}}({\text{g}}) = {\text{Post}}\;{\text{oven}}\;{\text{dried}}\;{\text{biomass}} + {\text{vial}}\left( {\text{g}} \right) - {\text{Pre - oven}}\;{\text{dried}}\;{\text{vial}}\;{\text{weight}}\left( {\text{g}} \right).$$

DCW values are typically used directly in microalgal species containing ash levels < 10%. However, diatoms typically contain considerable mineral ash because of the contribution of the non-carbonaceous silica exoskeleton^23^, although even green algae can exhibit ash content of 10% or more^24^. As a result, in species with higher ash percentages, the Ash Free Dry Weight (AFDCW; g/L) was determined to ensure accurate measurement of the concentration and productivity of individual biochemical constituents, since compositional analysis solely based on DCW measurements can underestimate the actual amount^25^. For AFDCW measurement, dry biomass was added to pre-weighed aluminum weigh pans and then reweighed. The pans were then placed in a muffle oven and oxidized (ashed) at 475 °C for 5 h. After cooling, the samples were reweighed and recorded. AFDCW% was calculated in accordance with the following equation:

$$\begin{aligned} {\text{AFDCW}}\left( \% \right) & = 100 \times \left( \left( \left( {{\text{Pre-combustion}}\;{\text{biomass}} + {\text{pan}}\left( {\text{g}} \right)}\right) \right. - \left( {{\text{Post-combustion}}\;{\text{biomass}} + {\text{pan}}\left( {\text{g}} \right)} \right) \right) \left. { /{\text{Pre-combustion}}\;{\text{biomass}}\left( {\text{g}} \right)} \right). \\ \end{aligned}$$

Because of the aforementioned high-ash concerns, AFDCW was used throughout this study and determined in accordance with the following equation:

$${\text{AFDCW}}\left( {\text{g}} \right) = {\text{DCW}}\left( {\text{g}} \right)*\left( {1 - {\text{AFDCW}}\left( \% \right)/100} \right).$$

#### Determination of biochemical composition

For each experimental setup, the total lipid content, total carbohydrate content, and total protein content were measured using previously published protocols^26^. Briefly, cell disruption was done mechanically with liquid nitrogen followed by the addition of chloroform/methanol (2:1 v/v) using the modified Bligh and Dyer method for lipid fractionation and separation. Total lipids were extracted from the chloroform phase, vacuum dried, and measured gravimetrically. Total carbohydrates were measured using the phenol sulfuric acid method with acid-hydrolyzed biomass, and total protein was calculated as the remainder from 100% after the % lipid, % carbohydrate, and % ash was subtracted^27,28^. Biomass productivity, lipid content, and lipid productivity were calculated using the formulas below:

$${\text{Biomass}}\;{\text{productivity}}\left( {{\text{mg}}/{\text{L}}\,{\text{d}}} \right) = AFDCW\left( {{\text{g}}/{\text{L}}} \right)/{\text{cultivation}}\;{\text{time}}\left( {days} \right).$$

$${\text{Lipid}}\;{\text{content}}\left( \% \right) = 100 \times \left( {{\text{Total}}\;{\text{lipids}}\;\left( {\text{g}} \right)/{\text{AFDCW}}\left( {\text{g}} \right)} \right).$$

$${\text{Lipid}}\;{\text{productivity}}\left( {{\text{mg}}/{\text{L}}\,{\text{d}}} \right) = {\text{Biomass}}\;{\text{productivity}}\left( {{\text{mg}}/{\text{L}}\,{\text{d}}} \right) \times \left( {{\text{Lipid}}\;{\text{content}}\left( \% \right)/{1}00} \right).$$

#### Gas chromatography–mass spectrometry quadrupole time-of-flight (GC–MS QTOF) analysis of fatty acid methyl esters (FAME)

Sample preparation was performed using standard protocols^29^ and FAME determination followed previously published protocols^30^. More specifically, total lipids extracted (5–10 mg) from algal cells were dissolved in chloroform, transferred to a clear glass vial, and then 0.6 M Methanolic HCl with C13 was added as internal standard to the glass vials, which were sealed and heated at 85 °C for 1 h for the transesterification reaction to occur. The vials were then cooled to room temperature, sealed, and stored in a freezer at − 20 °C until FAME extraction occurred using hexane by GC–MS analysis (Agilent Technologies 7200 GC–MS). The FAME composition was identified using the NIST (National Institute of Standards and Technology) mass spectral standard reference database. The identification and quantification of FAME was done by analyzing retention times and using library search reports (NISTIL.S database).

#### Liquid chromatography-mass spectrometry triple quadrupole (LC–MS QQQ) and spectrophotometric analysis of fucoxanthin

For fucoxanthin extraction from *C. cryptica*, 50 mg of lyophilized biomass were crushed using liquid nitrogen followed by extraction of carotenoids using ethanol (100%). The cell suspension was transferred to a glass vial and incubated overnight at 4 °C in the dark. The process was repeated until the cell pellet turned colorless. The ethanol extracts were then vacuum dried and re-suspended in 1 mL methanol for LC–MS using Agilent technologies 6460 Triple Quadrupole (QQQ) (Agilent 1100 Series, K1260 Infinity (2) stack). The fucoxanthin separation and determination were carried out based on a previously published protocol^30^. The mobile phase consisted of methanol and water with a flow rate of 0.400 mL/min at 35 °C with gradient elution in a C18 reverse phase column Eclipse Plus (Agilent, Santa Clara, CA USA) and a chromatogram recorded at 445 nm. Fucoxanthin standard (Catalog Number F6932-10 mg, batch number MKCP1541, Sigma-Aldrich, St. Louis, MO, USA) was used to both confirm the presence and location of the eluted precursor ion fragments and to construct the calibration curve across a concentration range of 1–10 µg/mL (Supplementary data).

The LC–MS results were also compared to spectrometric measurements. For spectrophotometric analysis, the carotenoids were dissolved in 1 mL hexane, and absorbance was read at 663 nm and 445 nm. Fucoxanthin concentration was the calculated by using a fucoxanthin calibration curve (0.2–10 µg/mL) (Supplementary data). The fucoxanthin absorbance at 445 nm was corrected by subtracting absorbance at 663 (chlorophyll interference). This correction is required because the calibration curve is only for fucoxanthin and there is possible overlap between the Chlorophyll (A and C at 663) and Fucoxanthin (at 445) absorptions.

### Statistical analysis

All the experiments were carried out in duplicate with the mean value ± standard deviation (S.D.) being reported. The data were plotted using Graph Pad Prism v 9.4.

## Results and discussion

### Determining gas exchange in AC-PBR

To determine the gas exchange in the AC-PBRs, the CO_2_ and O_2_ diffusion rates across the LDPE plastic were qualitatively estimated. The AC-PBR filled with CO_2_ (300 mL) was flat after ~ 24 h confirming the gradient diffusion of the enclosed CO_2_ to the outside air. Moreover, the AC-PBR submerged in water with an initial pH of 7.1 was also flat after ~ 24 h and the water pH lowered to 5.7, thus confirming again gradient diffusion of the enclosed CO_2_ into the water and subsequent formation of carbonic acid (H_2_CO_3_). In order to determine if there was any directionality on the in-air transfer, a separate, as-received air-filled AC-PBR was placed in a sealable, wide-mouthed, glass jar with the jar filled with CO_2_ and closed. The next morning, similarly, after ~ 24 h, the height of the AC-PBR was found to have swollen by ~ 4 times to a diameter larger than the mouth of the jar, confirming diffusion under a gradient of the outer CO_2_ outside through the LDPE AC wall into the inner air-filled region.

Similarly, the AC-PBRs filled with pure O_2_ was found to be smaller in volume after ~ 24 h than originally, but not nearly as flat as in the prior CO_2_ test, confirming the diffusion occurrence of the enclosed O_2_ to the outside air under a gradient, but at a much lower rate than CO_2_. The submerged AC-PBR also appeared to have slightly less volume with the total water volume reduced to ~ 2.95 L, also confirming the diffusion occurrence of the enclosed O_2_ to the outside water under a gradient but at a much lower rate than CO_2_. One thing to note was the presence of many small bubbles attached to the surface of the AC-PBR that were present in the water-submerged O_2_ test but were not present in the water-submerged CO_2_ test.

In conclusion, both CO_2_ and O_2_ were observed to diffuse across the AC-PBR surface under a positive gradient with CO_2_ diffusing at a much higher rate. This observation is supported by literature, as reported by Guisheng^31^, who cited relative values of permeability for CO_2_ and O_2_ of 10.7 and 3.1, respectively, for LDPE at 30°C^32^. The above findings complimented Khan’s results^19^ who filled wide-mouth glass jars with pond water and sealed them with a single sheet of AIRplus AC plastic film stretched across the jar rim and secured with two rubber bands. Results after 50 days revealed that the algae remained green with no observed loss in water volume, confirming that the algae present had experienced a sufficient exchange of gases (CO_2_ and O_2_) needed for photosynthesis and respiration. Overall, these combined positive results justified continuing on with the algae cultivation study in ACs during which any deleterious effects on growth duration and/or performance by O_2_ toxicity build-up and water loss would be monitored.

Even though we observed efficient gas transfer via the AC-PBR surface, to ensure that CO_2_ is present throughout the cultivation period, the respective media for each algal strain was supplemented with 48 g/L of NaHCO_3_ in addition to bubbling of CO_2_. This established our CO_2_-bicarbonate buffer system^33^ which significantly increased the O.D._680 nm_ by nearly fourfold for *C. vulgaris and* nearly eightfold for *N. oculate* over non-CO_2_ enhanced fresh and marine media (data not shown)*.* Based on these observations, the procedure of bubbling in CO_2_ followed by sodium bicarbonate addition to a neutral pH was made part of the baseline configuration for all species tested, including the diatom *C. cryptica*.

### Algal growth rate in AC-PBRs

To examine the applicability of reused and repurposed plastic AC-PBRs for microalgal cultivation, we selected three fresh and marine algae strains: *C. vulgaris*, *N. oculata*, and *C. cryptica*. *C. vulgaris* is a freshwater model green microalga often used as a dietary supplement or protein-rich food additive and is also reported to accumulate high biomass and intracellular lipid content^33^. *N. oculata* is a photoautotrophic, unicellular, free-floating marine green alga often used in aquaculture as an energy-rich food source for fish larvae and rotifers^34,35^. In addition, it is considered a promising alga for industrial and nutraceutical applications because of its ability under proper cultivation conditions to accumulate high levels of polyunsaturated fatty acids (PUFA) and carotenoids, such as astaxanthin, zeaxanthin, and canthaxanthin. *C. cryptica* is a photo/heterotrophic unicellular, free-floating, centric marine diatom (brown alga) often used in aquaculture feeds^36^. It is considered a promising alga for industrial and nutraceutical applications because of its ability to accumulate high levels of PUFA and the carotenoid fucoxanthin^37,38^. In addition, since diatoms are characterized by an almost pure, amorphous hydrated silica shell (frustule), they can also serve as a potential source of biogenic silica for high quality applications^39,40^. Finally, *C. cryptica* is also known to produce β-chitin nanofibrils (fibers)^3,41^, which may have use in the manufacture of biomembranes and bioplastics that have better biodegradability than plastics^42–44^. The production of biobased plastics from natural resources with high levels of protein and carbohydrate-based polymers present a biodegradable alternative for replacing or complementing traditional petroleum-based plastics^45,46^.

**Figure 1:**
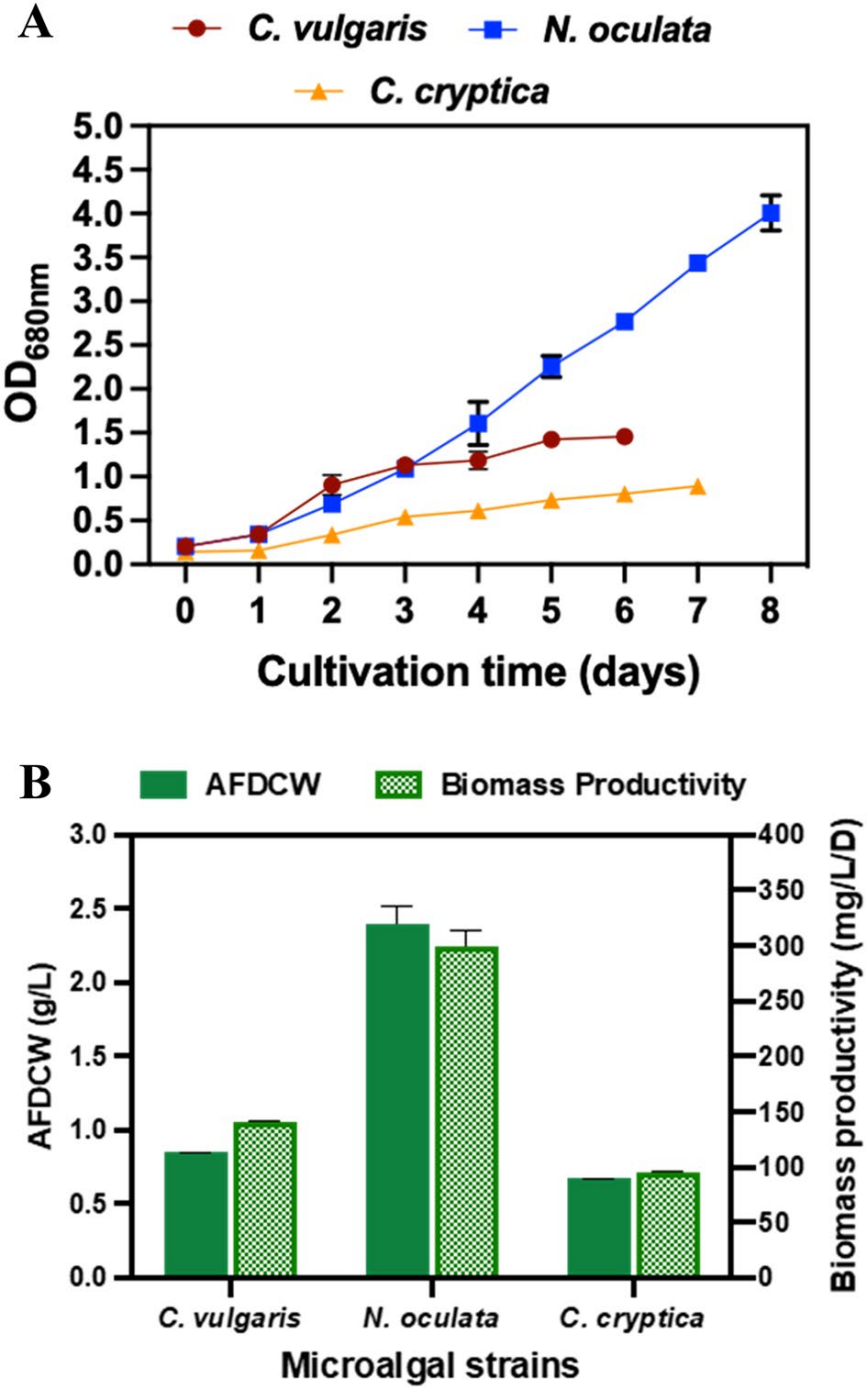
(**A**) Growth curve (O.D._680 nm_); and (**B**) Ash-free dry cell weight (AFDCW; g/L) and biomass productivity (mg/L d) of *C. vulgaris*, *N. oculata*, and *C. cryptica* cultivated in AC-PBR.

Notably, all three algal strains were able to adapt and grow in AC-PBRs with *N. oculata* showing the highest level of growth as it attained an O.D._680 nm_ of 4.01 ± 0.20 followed by *C. vulgaris* and *C. cryptica* (Fig. 1A). The algal strains displayed a 1-day lag phase (0–1 day) followed by growth between days 2 and 5. According to the growth curves, *C. vulgaris* reached early stationary phase (harvesting time) on the 6th day, while *N. oculata* and *C. cryptica* continued to grow until the 8th and 7th days, respectively (Fig. 1A). Approximate measurement of residual nitrate and phosphate revealed exhaustion of these two macronutrients in *C. vulgaris* cultures on the 6th day, while *N. oculata* and *C. cryptica* cultures had 250 mg/L and 10 mg/L of residual nitrate and phosphate on the 8th and 7th days, respectively, explaining the longer duration of growth. Since it is known that certain marine algae and diatoms contain minerals and silica at 22–47% of the total biomass, we adjusted the DCW for all the algal strains to avoid overestimation of biomass. A review of the literature revealed low ash levels for *C. vulgaris* at approximately ~ 5–6%^47,48^, so in accordance with common research practices no adjustment pertaining to % ash was made herein (i.e. AFDCW (g) = DCW (g)). A review of the literature for *N. oculata* and *Nannochloropsis sp.* revealed consistent ash levels at 24.5 and 27.3%^49,50^, respectively; therefore, for this analysis, an average value of 25.9% ash content was used herein. Examining the literature for diatoms revealed that Hildebrand^26^ compared eight different diatom species with ash contents from 49 to 59%, with diatoms having on average twice the ash content of a variety of non-silicified algae and four times the content of two *Chlamydomonas* species examined. Because of the larger magnitude value and variation in silica content noted for diatom species calculation of the organic content of dry weight, direct duplicate measurements of AFDCW% were made for *C. cryptica* using the literature mean ash content value of 45.4%.

Overall, the highest AFDCW and biomass productivity of 2.39 g/L and 298.55 mg/L/day was attained by *N. oculata*, followed by *C. vulgaris* at 0.85 g/L and 141.36 mg/L/day, and *C. cryptica* at 0.67 g/L and 96.08 mg/L/day (Fig. 1B). Baseline published algal biomass productivity values for traditional RWP and PBR typically fall within the ranges of 0.5–1 g/L and 2–6 g/L, respectively^51^. Hence, the observed AC-PBR biomass productivity of the algal strains was comparable to those reported for indoor PBRs and RWPs (Table 1)^52–54^, rendering credence to the potential of cost-effective use of ACs as PBRs that would serve as a “set and forget” nutrient-replete algal cultivation system.

**Table 1:** Comparison of biomass and constituent productivity of microalgal strains cultivated in AC-PBRs in this study versus performance in common PBRs as reported in the literature.

Microalgae strain	Cultivation method	Biomass productivity (mg/L/d)	Lipid productivity (mg/L/d)	Carbohydrate productivity (mg/L/d)	Protein productivity (mg/L/d)	References
*Chlorella vulgaris*	Indoor photo-bioreactor (PBR)	121.93	26.21	14.63	29.99	^52^
*Nannochloropsis oculata*	Indoor open raceway pond (RWP)	210	113.06	–	–	^53^
*Cyclotella cryptica* UTEX 1269	Indoor carboy container	175.83	29.01	62.94	45.71	^54^
*C.vulgaris*	AC-PBR (AFDCW)	141.36	24.96	22.61	93.80	This study
*N.oculata*		298.55	20.35	30.78	247.42	
*C.cryptica*		96.08	25.54	53.69	16.85	

### Biochemical analysis of the algal biomass

**Figure 2:**
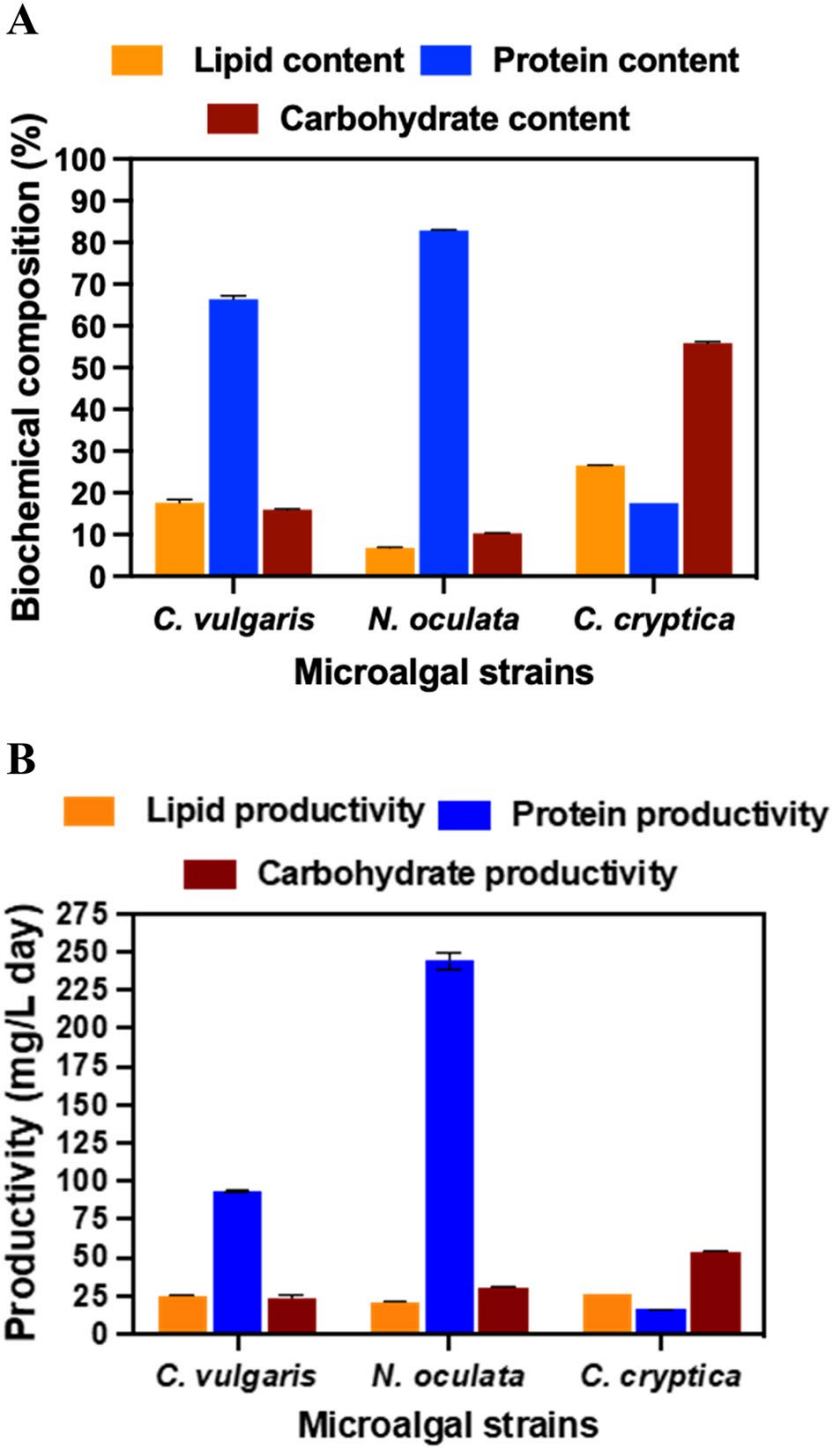
(**A**) Biochemical composition (%); and (**B**) productivity (mg/L d) of *C. vulgaris*, *N. oculata,* and *C. cryptica* cultivated in AC-PBR.

Depending upon the species, microalgal biomass is primarily composed of carbohydrates (4–64%), lipids (4–45%), and proteins (6–71%)^55^. As stated earlier, inclusion of ash content in the biochemical composition can lead to overestimation of the macromolecules, thus the total protein, total carbohydrate, and total lipids along with their respective productivities were calculated on an AFDCW basis (Fig. 2A,B). The maximum lipid content (26.58 ± 0.30% AFDCW) was achieved in *C. cryptica,* followed by C*. vulgaris* at 17.66 ± 0.30% AFDCW and *N. oculata* at 6.82 ± 0.21% AFDCW (Fig. 2A). Similarly, the maximum lipid productivity (25.54 ± 0.33 mg/L/day AFDCW) was achieved by *C. cryptica,* followed closely by *C. vulgaris* at 24.96 ± 0.31 mg/L/day and then *N. oculata* at 20.35 ± 0.51 mg/L/day (Fig. 2B). It should be noted that *C. vulgaris,* which exhibited a lower biomass concentration than *N. oculata* and a higher biomass concentration than *C. cryptica,* had a lipid productivity not significantly different than either one (Fig. 2B). As mentioned in the previous section, nitrate was completely depleted in *C. vulgaris* cultures, while *N. oculata* modified F/2 medium and *C. cryptica* L1 medium had some residual (50–250 mg/L) nitrate at the end of the cultivation cycle. Indeed, previous studies have reported nitrogen starvation to trigger biosynthesis of intracellular lipids by several algae. Hence, increase in the lipid content of the algae species could be attributed to nitrogen depletion^56,57^.

It has been reported that algae initially synthesize carbohydrates under early nutrient stress followed by a switch to lipid biosynthesis, when the stress is prolonged^58^. Because of the large amount of silica present in the *C. cryptica* diatom biomass, we were unable to use the standard phenol sulfuric acid method and Bradford assay to measure total carbohydrate and protein content, respectively, and hence opted for elemental analysis (CHNS). The protein content in the diatom was estimated by the Nitrogen to Protein (NtP) conversion factor. The NtP conversion value often used is 6.25 based on cereals using a common elemental composition of C_40_H_62_N_10_O_12_ for protein^59^, with the nitrogen content of the formula being 16% and its reciprocal (1/0.16) equaling 6.25^60,61^. However, later research indicated that this value may be too high and that an average factor of 4.78 should be used for algal biomass, with 4.68 measured for the diatom *Phaeodactylum tricornutum*^62^. The CHNS analysis data showed % N of 2.05 ± 0.07 in the *C. cryptica* biomass corresponding to a total protein content of 9.6% of DCW. The AFDCW carbohydrate content was then calculated by subtracting the total AFDCW lipid and the total protein AFDCW content. The highest AFDCW-based carbohydrate productivity was observed in *C. cryptica* at 53.69 ± 0.19 mg/L/day, followed by *N. oculata* at 30.78 ± 1.81 mg/L/day and *C. vulgaris* at 22.61 ± 2.72 mg/L/day (Fig. 2B). The highest AFDCW based protein productivity was observed in *N. oculata* at 247.42 ± 14.16 mg/L/day, followed by *C. vulgaris* at 93.80 ± 1.74 mg/L/day and *C. cryptica* at 16.85 ± 0.03 mg/L/day (Fig. 2B). All taken together, the biochemical productivity values of proteins, lipids, and carbohydrates are comparable with those reported in literature for these strains cultivated in indoor PBRs, reinforcing the potential of AC-PBR as a low-cost sustainable cultivation system that contributes to plastic reuse (Table 1).

### Fatty acid methyl ester (FAME) profile analysis

**Figure 3:**
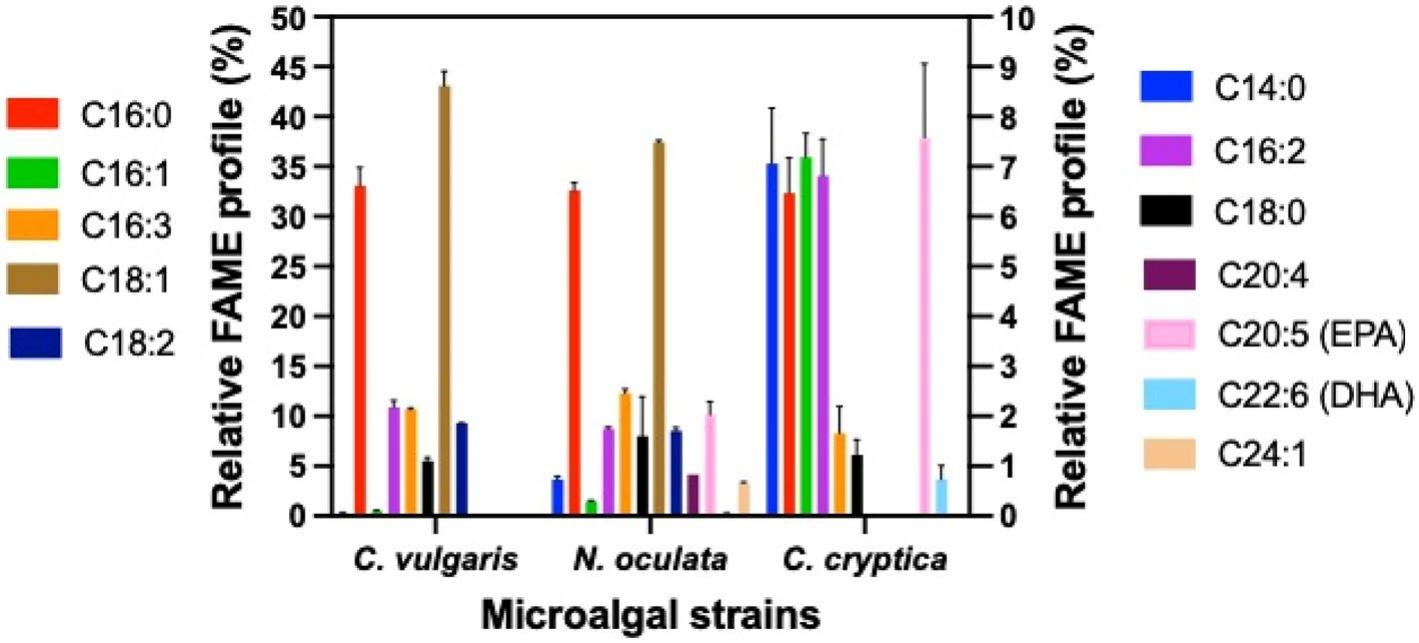
Relative FAME profile (%) of *C. vulgaris*, *N. oculata,* and *C. cryptica* cultivated in AC-PBR.

Determination of the FAME profile of *C. vulgaris*, *N. oculata*, and *C. cryptica* cultivated in AC-PBRs was performed to investigate changes in the fatty acid profile. The *C. vulgaris* FAME profile was found to comprise mainly palmitic acid (16:0), hexadecatrienoic acid (16:3), oleic acid (18:1), linoleic acid (C18:2), and stearic acid (C18:0), which is in line with previously published studies^6,58^ (Fig. 3). Saturated fatty acids (SFA, no double bonds), monounsaturated fatty acids (MUFA, one double bond), and polyunsaturated fatty acids (PUFA, two or more double bonds) accounted for 34.25%, 43.62%, and 22.14% of total fatty acid content, respectively. As noted earlier, nitrate was depleted in *C. vulgaris* cultures apparently causing the observed accumulation of C18:1, which plays a vital role in quenching reactive oxygen species generated during nutrient deprivation^26^. Moreover, higher MUFA and PUFA content is favored under nutrient depletion since they are precursors for membrane lipid biosynthesis and aid in membrane modulation. The *N. oculata* FAME profile mainly comprised C16:0, C16:3, C18:1, and C18:2, with small amounts of eicosapentaenoic acid (EPA) C20:5 and C24:1 (Fig. 3). SFA, MUFA, and PUFA accounted for 34.96%, 39.57%, and 25.47% of total fatty acids, respectively. These results are in line with the previously reported FAME profile for *Nannochloropsis* sp.^30,34^. The membrane lipids of *N. oculata* contain long chain (LC) PUFA, including EPA, C20:4, and C24:1, which contribute to the presence of these fatty acids in the algal biomass. Lastly, the *C. cryptica* FAME profile mainly comprised C14:0, C16:0, C16:1, C16:2, C16:3, C20:5, and docosahexaenoic acid (DHA) C22:6 (Fig. 3). SFA, MUFA, and PUFA accounted for 40.63%, 35.94%, and 23.43% of total fatty acids, respectively. These findings are consistent with diatom-related content found in the literature, including the presence of DHA (0.74%), relatively high levels of EPA (7.58%), and trace levels of C18 fatty acids^63,64^.

In end use applications, MUFA produce better biodiesel, when considering low temperature fluidity and oxidative stability, whereas PUFA, like DHA and EPA, have been shown to be beneficial to human health with end products used in the pharmaceutical and cosmetic industries, as well as in dietary supplements for the carotenoid fucoxanthin. All three strains exhibited comparable lipid productivity for biofuel, nutraceutical, and bioplastic production via the protein-rich *C. vulgaris*^44,65^ and *N. oculata* or the β-chitin nanofibrils of *C. cryptica.* Moreover, depending upon the co-product extraction process used, the remaining algal biomass, which is rich in protein, could serve as a feedstock to augment animal and aquaculture feed, as well as contribute to human nutritional needs caused by increases in population, changes in climate, and the declining availability of arable land for farming^42,66^.

### Fucoxanthin analysis

**Figure 4:**
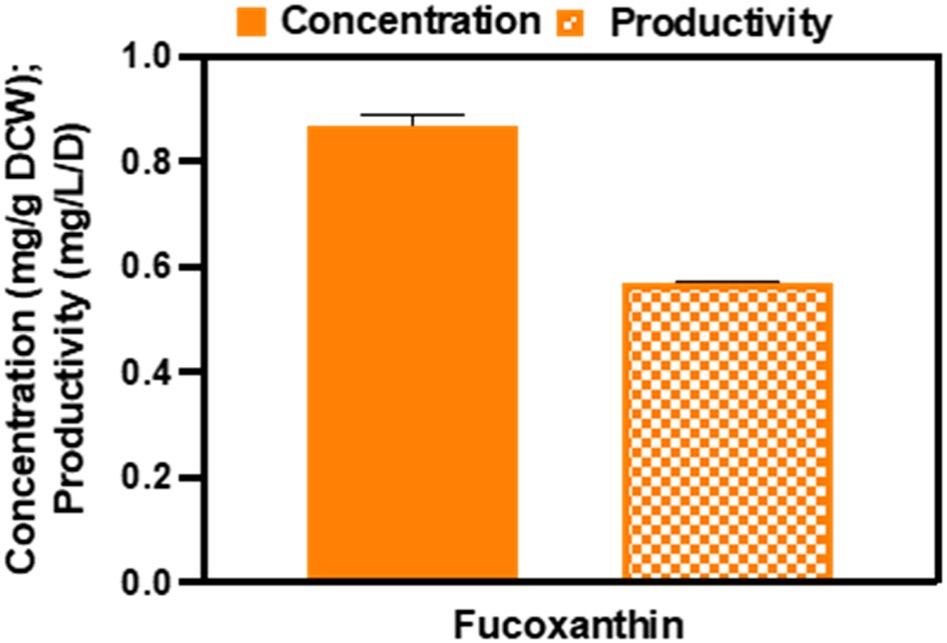
Fucoxanthin concentration (mg/g DCW) and productivity (mg/L d) in *C. cryptica* cultivated in AC-PBR.

Fucoxanthin is a major marine carotenoid that occurs abundantly in both macroalgae (seaweed) and microalgae contributing to approximately 10% of the estimated total production of carotenoids in nature^67,68^. In microalgae, especially in diatoms like* C.* cryptica, orange-colored fucoxanthin (C_42_H_58_O_6_) is one of the main photosynthetic cell pigments and non-provitamin A carotenoids used by the cell to harvest light and transfer energy^69,70^, with the other two major photosynthetic light-harvesting pigments being chlorophyll *a* (chl *a*), and chlorophyll *c* (chl *c*)^8^. In recent years, fucoxanthin has been studied as a dietary supplement in terms of its anti-inflammatory, anti-tumor, anti-obesity, anti-diabetes, and anti-malarial therapeutic activities^71^. Currently, fucoxanthin is mainly produced from slow-growing, low fucoxanthin content seaweed. Faster growing microalgae are an important alternative option for fucoxanthin production because of their easier cultivation and scalability characteristics^72^. The LC–MS analysis of *C. cryptica* cultivated in AC-PBR showed a fucoxanthin concentration of 0.868 mg/g DCW (0.474 mg/g AFDCW) and a fucoxanthin productivity of 0.570 mg/L d (0.311 mg/L d AFDCW) (Fig. 4). The LC–MS results were comparable with the spectrophotometric fucoxanthin concentration results (0.822 mg/g DCW), which proved to be a much simpler and faster method for detecting fucoxanthin in diatom samples. The calculated DCW fucoxanthin productivity value of 0.570 mg/L d was more than double the upper range value of ~ 0.23 mg/L d for 13 diatoms, including *C. cryptica* (CCMP 333; ~ 0.12 mg/L d) cultivated in Erlenmeyer flasks in modified SK medium at a lower light intensity of 30 µmol/m^2^s and a longer cultivation period of 14 days^37^.

## Conclusion

Cultivation of microalgae towards a single bioproduct has found limited economic success to date. This realization is driving a shift in the focus of the entire enterprise towards a multi-product biorefinery structure in which targeted products, co-products, and system improvements in design contribute positively to the overall success of the operation by improving the bottom-line process economics. In this study, we performed a laboratory assessment of ACs as low-cost and low-labor PBRs to grow *C. vulgaris, N. oculata*, and the diatom *C. cryptica*. Repurposed and reused ACs (1) Reduce and mitigate plastic manufacture and waste contributing to sustainability efforts; (2) Reduce biorefinery capital investment; and (3) Provide an isolated, contaminant-free environment during algal growth and bioproduct synthesis. The generated data compared favorably with traditional PBR values found in the literature for algal biomass, lipid, and fucoxanthin productivity, thus forming a reference point for other polyethylene-based plastic bag PBRs of different design and configurations under consideration. Ultimately, the applicability and utility of ACs will depend upon the degree of optimization of the developed products, utilized scale, and overall economics. Several direct use applications are envisioned for AC-PBRs, including manufacturing of high-value products/molecules and investigation of alternate cultivation pathways, such as microalgal biofilm cultivation.

## Supplementary Information

Supplementary Information.

## Data Availability

The datasets used and/or analyzed during the current study are available at the Box.com online data repository, https://usf.app.box.com/s/3nctk2w36hwn7cjwqk7v4a4lpnxduuv6.
