# Supplementary material for: Microalgal cultivation characteristics using commercially available air-cushion packaging material as a photobioreactor
Source: Sci Rep. 2023 Mar 7;13:3792. doi: 10.1038/s41598-023-30080-6 (PMC9992509; doi:10.1038/s41598-023-30080-6)

**SUPPLEMENTARY DATA**

**Microalgal Cultivation Characteristics Using Commercially Available Air-Cushion Packaging Material as a Photobioreactor**

**Clifford R. Merz^1*^, Neha Arora^2^, Micheal Welch^3^, Enlin Lo^3^, George P. Philippidis^3^**

^1^College of Marine Science, University of South Florida, St. Petersburg, FL, USA

^2^Department of Cell, Microbiology and Molecular Biology, University of South Florida, Tampa, FL, USA

^3^ Patel College of Global Sustainability, University of South Florida, Tampa, FL, USA

* Corresponding author. Tel: +1-727-553-3729; Fax: +1-727-553-1189 E-mail address: [cmerz@usf.edu](mailto:cmerz@usf.edu)

**Supplementary Figure 1: (A)** Microalgal cultivation AC-PBR experimental set-up **(B)** Cultures of *C. vulgaris* (lighter green, top), *N. oculata* (darker green, bottom) and *C. cryptica* (brown, bottom) cultivated in 1/2 full AC-PBR

**A**


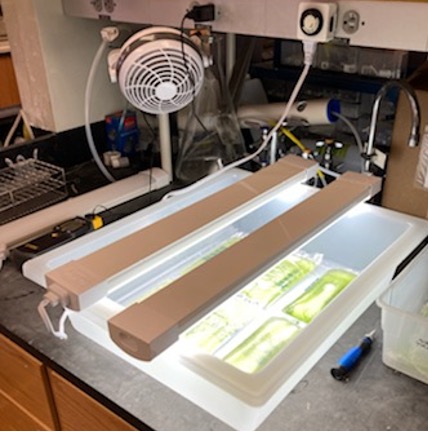


**B**


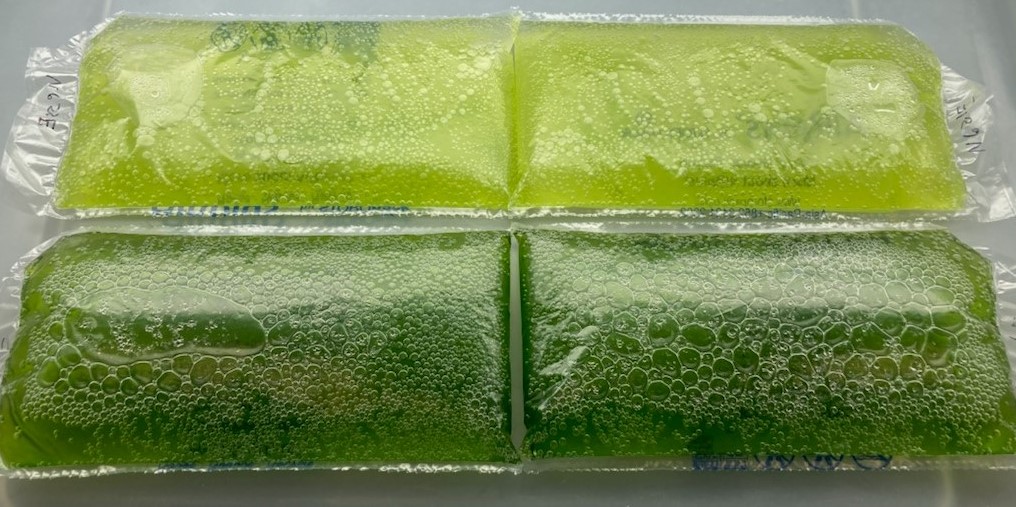


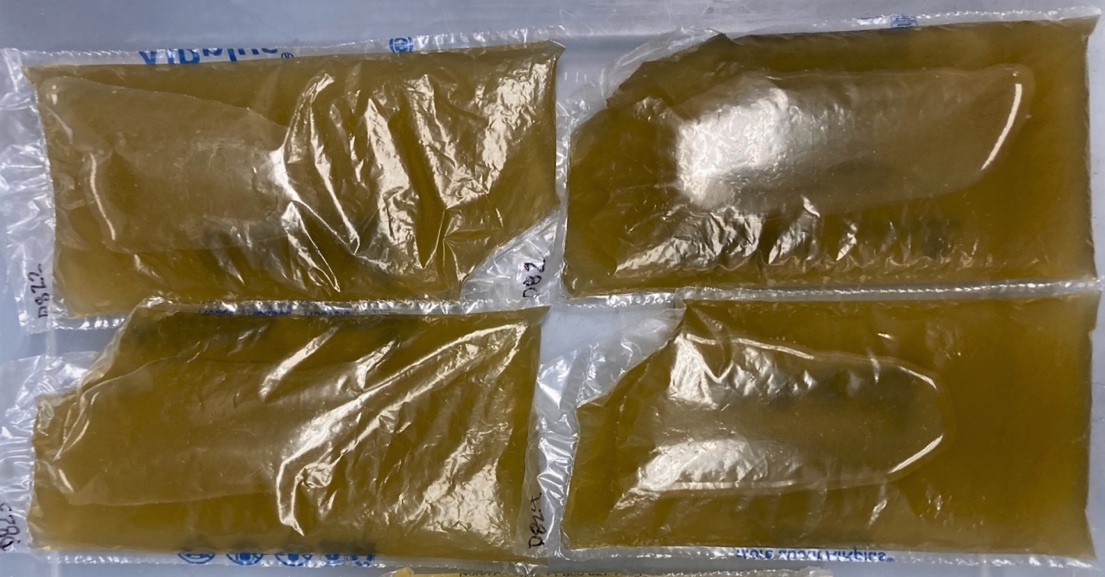


**Supplementary Figure 2:** Growth curve (O.D._680nm_) of *C. vulgaris* and *N. oculata* cultivated in ¼ and ¾ full AC-PBR in BBM and modified f/2, respectively.


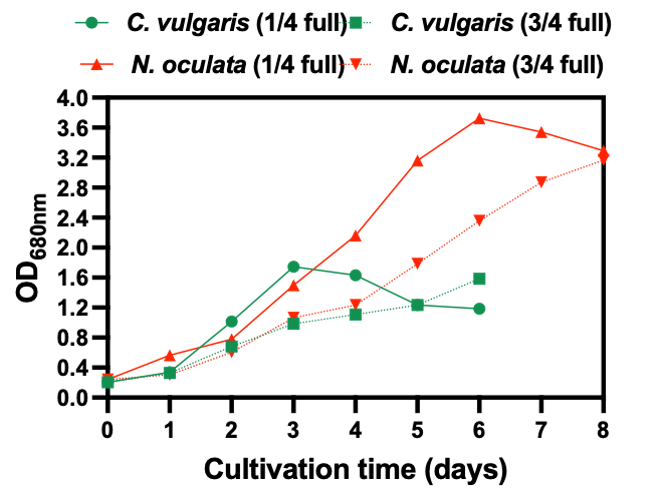


**Supplementary Figure 3:** Standard curve of fucoxanthin (1 to 10µg/mL) for LC-MS analysis of fucoxanthin in *C. cryptica.*


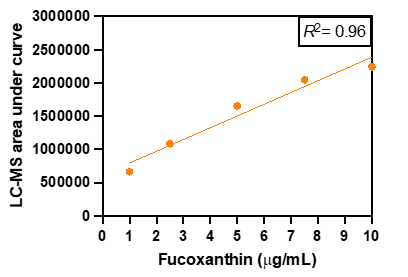


**Supplementary Figure 4:** Standard curve of fucoxanthin (0.2 to 10µg/mL) for spectrophotometric analysis of fucoxanthin in *C. cryptica.*


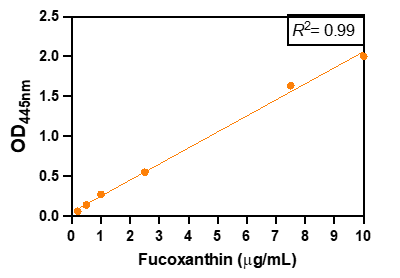

Supplement: Supplementary file 1 — Supplementary Information. [file 41598_2023_30080_MOESM1_ESM.docx]
